# Prevalence of glucose-6-phosphate dehydrogenase deficiency in Cameroonian blood donors

**DOI:** 10.1186/s13104-019-4226-z

**Published:** 2019-04-02

**Authors:** Stephanie M. Lauden, Stella Chongwain, Anzeh Achidi, Ethan Helm, Sarah E. Cusick, Amelia Krug, Tina M. Slusher, Troy C. Lund

**Affiliations:** 1 Nationwide Children’s Hospital, The Ohio State University, Columbus, OH USA; 2 Mbingo Baptist Hospital, Mbingo, Cameroon; 3 Division of Global Pediatrics, Department of Pediatrics, University of Minnesota, Minneapolis, MN USA; 4 Global Pediatrics, Stem Cell Institute, Pediatric Blood and Marrow Transplant Program, University of Minnesota, MMC 366, 420 Delaware St SE, Minneapolis, MN 55455 USA

**Keywords:** Africa, G6PD deficiency, Blood donor

## Abstract

**Objective:**

Deficiency in G6PD is the most common enzymopathy worldwide. It is frequently found in individuals of African descent in whom it can lead to hemolytic crises triggered by the use of certain antimalarial medications and infection. The prevalence of G6PD deficiency and its contribution to morbidity in West Africa is under-studied. To understand the prevalence of glucose-6-phosphate dehydrogenase (G6PD) deficiency in West African blood donors.

**Results:**

We evaluated the G6PD status and infectious disease screening tests of 1001 adult male Cameroonian blood donors (mean age 31.7 ± 9.8 years). The prevalence of G6PD deficiency was 7.9%. There was no difference in levels of hemoglobin or ABO subtype between those who were G6PD-normal compared to those that were deficient. Interestingly, G6PD-normal vs. deficient blood donors were less likely to have screened positive for hepatitis C virus (p = 0.02) and rapid plasma reagin (indicative of syphilis, p = 0.03). There was no significant difference in hepatitis B sAg, HIV-1, or HIV-2 reactivity between those with vs. without G6PD sufficiency. These data suggest that G6PD deficiency is common among West African male blood donors and may be associated with specific infectious disease exposure.

## Introduction

Glucose-6-phosphate dehydrogenase (G6PD) deficiency is the most common human enzymopathy, affecting upwards of an estimated 400 million people worldwide [1, 2]. Population screening in regions where the prevalence of G6PD deficiency is 3–5% or greater (in males) is recommend by the World Health Organization (WHO) [3]. Due to cost and lack of infrastructure, this has yet to become regular practice in many parts of the world. The regional prevalence of G6PD deficiency in Africa, ranges from 15 to 26% [4]. G6PD deficiency contributes to hyperbilirubinemia and jaundice in newborns, putting infants at risk for acute bilirubin encephalopathy within the first few days of life that may result in subsequent death or kernicterus spectrum disorder. Kernicterus spectrum disorder is manifest by hearing deficits, behavior problems, and long-lasting neurologic damage [5]. G6PD deficiency can also cause morbidity in persons receiving antimalarials such as dapsone or primaquine, by causing hemolysis and hemoglobinuria [6, 7]. These factors contribute to the overall public health burden in this condition.

Glucose-6-phosphate dehydrogenase deficiency is reported to confer partial resistance to malaria in deficient males as well as in heterozygous females, explaining its higher prevalence in malaria-endemic regions [1, 8, 9]. In this study, we determined the prevalence of G6PD deficiency in over 1000 male Cameroonian blood donors using the fluorescent spot test. Donors also underwent standard screening for syphilis, hepatitis B virus (HBV), hepatitis C virus (HCV), HIV-1, and HIV-2. We evaluated the donor screening results for associations with G6PD deficiency.

## Main text

### Subjects studied

We assessed the prevalence of G6PD deficiency and its association with infectious disease screening Cameroonian blood donors at Mbingo Baptist Hospital (Mbingo, Cameroon). Per the previously established Mbingo Baptist Hospital blood donation system, potential donors underwent a standardized donation process between January 2016 and July 2017. A standardized questionnaire eliminated donors with recent illness, recent blood donation, intravenous drug use, history of jaundice or hepatitis, history of known sexually transmitted infection, or known significant comorbidities (known blood disease, cancer, gastrointestinal bleeds, diabetes, thyroid disease, or kidney disease). Potential donors underwent a series of screening tests, including those for anemia and ABO blood typing. Donors were screened for infections using commercially available detection systems. Syphilis detection was done via rapid plasma reagin test (RPR, ACON Laboratories, San Diego, CA), hepatitis B surface antigen using an enzyme immunoassay (EIA, ACON Laboratories, Healthmate rapid hepatitis C viral antigen test (DFI Co. Ltd., Gimhae, Korea), HIV-1 and HIV-2 were determined using Alere Determine (Waltham, MA) or First Response HIV1-2-0 (Premier Medical Corporation, Denver, CO). ABO testing was performed via standard serological reactivity assays.

### Glucose-6-phosphate dehydrogenase assay

Approximately 200 µL of whole blood from each donor was frozen within 4 h of collection at − 70 °C in a low temperature freezer which was monitored daily. Samples were thawed one time, which was at the time of assay. G6PD deficiency was determined using the Beutler fluorescent spot test [10]. The fluorescent spot test works on the principle that NADPH, which is produced from NADP in a reaction catalyzed by G6PD, fluoresces under long-wave ultra-violet light. A fluorescent spot indicates G6PD activity is present [10]. All samples were run in batch at the end of the study.

## Results and discussion

**Fig. 1:**
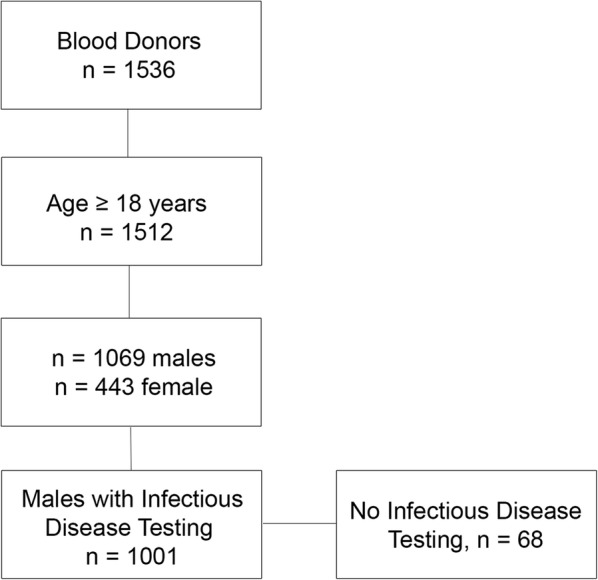
Flow diagram of blood donor screening

We screened a total of 1512 donors over 18 years and identified 7.1% as G6PD-deficient (Fig. 1). The cohort of blood donors was predominantly male (74%, n = 1001). It is well known that most all enzymatic tests for G6PD deficiency can give false negative results in females due to X-linked heterozygosity and random X inactivation [11, 12]. Therefore, the remainder of our analyses focused on the cohort of 1001 male blood donors. We found the prevalence of G6PD-deficiency in males to be 7.9%. This result is in line with historic publication of prevalence of G6PD deficiency in Cameroon with Bernstein el al reporting 6.4% and 5.7% [13, 14]. There have been mixed publications that show G6PD prevalence can decrease with age, suggesting reduced health fitness in those that are G6PD-deficient, though this has not been confirmed in large studies [11, 15, 16]. The mean age of G6PD-deficient blood donors in our study was 32.8 years compared to 31.6 years in G6PD normal donors (p = 0.27). Among G6PD-deficient donors, there was no difference in prevalence of deficiency by age quartile (Chi square p = 0.69, Table 1).

**Table 1:** G6PD prevalence in male Cameroonian blood donors

	n	G6PD deficient	Rate (%)
Cohort	1512	95	7.1
Males	1001	79	7.9
Males, 18–29.9 years	474	38	8.0
Males, 30–39.9 years	296	21	7.1
Males, 40–49.9 years	159	15	9.4
Males, 50–59.9 years	56	5	8.9
Males, > 60 years	16	0	0.0

**Fig. 2:**
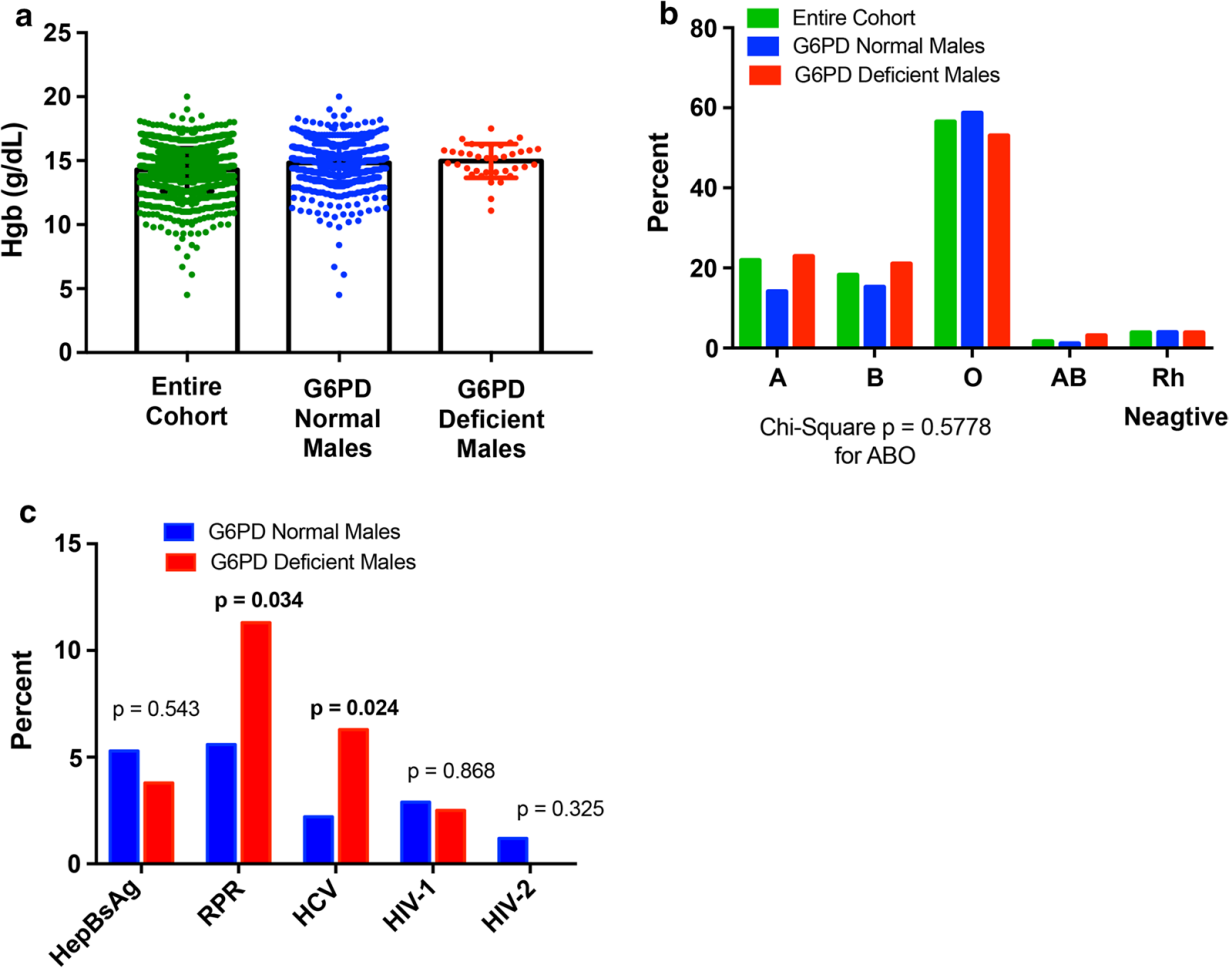
Hematologic parameters and infectious disease screening amongst G6PD normal and deficient blood donors. **a** Mean hemoglobin concentrations if Cameroonian blood donors. Show are the means and standard deviations. **b** Distribution of ABO blood type in Cameroonian blood donors. **c** Rate of infectious disease screen positivity in G6PD normal and deficient males Cameroonian blood donors. p-values were generated using a Chi square analysis

Evaluation of hemoglobin (Hgb) concentration between G6PD normal (14.8 g/dL) and G6PD-deficient donors (14.9 g/dL)(Fig. 2a, p = 0.52) revealed no significant difference. This may be expected given that the African G6PD variant (A−) is often a WHO class III (retaining 10–60% residual activity), and hemolysis and anemia are often preceded by a “trigger”, i.e. infection, medication, drugs, specific foods (fava beans being the prototype) [1]. There were also no differences in the distribution of ABO or Rhesus factor between deficient and normal groups (Fig. 2b).

Interestingly, infectious disease screening tests showed that G6PD-deficient donors had a significantly higher prevalence of hepatitis C virus (HCV) and rapid plasma reagin (RPR) positivity as compared to normal donors (p = 0.024 and 0.034, respectively, Fig. 2c). There was no difference in HepBsAg or HIV-1 reactivity. There were no HIV-2 positive, G6PD-deficient males. An association between syphilis and hepatitis C exposure has not been described in the literature [11], although two studies have shown an association between G6PD deficiency and increased clinical severity of hepatitis A and E [17, 18]. Whether this was related to G6PD-associated red cell fragility or an innate immune system defect was not known. Note, we determined that the mean Hgb for those with G6PD deficiency and HCV reactive was 15.1 g/dL versus with G6PD deficiency and HCV non-reactive was 15.2 g/dL (p = 0.90) consistent with a prior report that G6PD deficiency is not associated with an increased risk of hemolysis [19]. The mean Hgb for those with G6PD deficiency and RPR reactive was 15.3 g/dL versus G6PD deficiency and RPR non-reactive was 15.1 g/dL (p = 0.74).

The associations between G6PD and blood donation infection disease screening were surprising and previously undescribed in the literature. Though, there have been case reports and small case series that G6PD deficiency can exacerbate infections and clinical presentation [17, 18, 20–23]. For example, Au et al. and Gotsman et al. both reported more severe clinical presentations of patients with Hepatitis E virus (HEV) and Hepatitis A virus (HAV), respectively [17, 18]. Similarly, Jain et al. reported significantly higher levels of indirect bilirubin and longer hospital stay in patients with G6PD deficiency with acute viral hepatitis (HAV or HEV) compared to those that did not have G6PD deficiency [22]. While these cases represent increased severity of disease presentation, a mechanism of increased infection risk is not known.

Theoretically, the pathophysiology of G6PD deficiency may contribute to potential immune dys-regulatory relationships between G6PD deficiency and infection. The activity of G6PD is an essential step in the production of NADPH and reduced glutathione protecting erythrocytes from free radicals and associated oxidative stress. White blood cells also utilize NADPH-regulated pathways (among others) that contribute to the bactericidal respiratory burst. Prior studies have shown that leukocytes or neutrophils from patients with severe (class I) G6PD deficiency have decreased reactive oxygen species (ROS) production, potentially contributing to increased susceptibility to infectious disease [24, 25]. Supporting this mechanism, Siler et al. showed that in three siblings with severe G6PD deficiency, there was reduced NADPH oxidase activity in their granulocytes and reduced NETosis, contributing to susceptibility to bacterial infection [26]. It should be noted that the African type of G6PD deficiency if often a milder form (Class III) than the severe G6PD deficiency (Class I). Finally, Rostami-Far et al. similarly showed that the prevalence of G6PD deficiency in infants with sepsis (n = 76) was higher than that of non-septic neonates (n = 1214) at 10.9% and 2.9%, respectively (p = 0.03) [21]. Sepsis mostly consisted of Gram-positive bacteria in the G6PD deficient group (62.5%) while Gram negative bacteria were more common the G6PD normal group (55.9%).

## Limitations

Limitations include the fact that it was a cross-sectional study and from a single site where confirmatory testing of infections was not routinely performed. Additionally, testing is based on prior exposure and antibody responsiveness. An alternative explanation for the associations observed in this study could be a higher antibody response generated in G6PD deficient donors after exposure. For these reasons, our findings need to be repeated in other centers and populations to confirm validity but suggest a previously undescribed link between G6PD deficiency and infectious disease screening in adult male blood donors that may be important for future public health inquiries.

## Abbreviations

G6PD: glucose-6-phosphate dehydrogenase
WHO: World Health Organization
HIV: human immunodeficiency virus
HBV: hepatitis B virus
HCV: hepatitis C virus
RPR: rapid plasma reagin
EIA: enzyme immunoassay
ROS: reactive oxygen species
